# Comparative Assessment of Bacteriophage and Antibiotic Activity against Multidrug-Resistant *Staphylococcus aureus* Biofilms

**DOI:** 10.3390/ijms23031274

**Published:** 2022-01-24

**Authors:** Natalia Kaźmierczak, Bartłomiej Grygorcewicz, Marta Roszak, Beata Bochentyn, Lidia Piechowicz

**Affiliations:** 1 Department of Medical Microbiology, Faculty of Medicine, Medical University of Gdańsk, Dębowa 25, 80-204 Gdańsk, Poland; 2 Department of Laboratory Medicine, Chair of Microbiology, Immunology and Laboratory Medicine, Pomeranian Medical University in Szczecin, Powstańców Wielkopolskich 72, 70-111 Szczecin, Poland; bartlomiej.grygorcewicz@pum.edu.pl (B.G.); martaroszak95@gmail.com (M.R.); 3 Faculty of Applied Physics and Mathematics, Gdańsk University of Technology, Narutowicza 11/12, 80-233 Gdańsk, Poland; beata.bochentyn@pg.edu.pl

**Keywords:** bacteriophages, antibiotic, biofilm, phage therapy, antibiofilm, antibiotic resistance

## Abstract

Problems connected with biofilm-related infections and antibiotic resistance necessitate the investigation and development of novel treatment strategies. Given their unique characteristics, one of the most promising alternatives to conventional antibiotics are bacteriophages. In the in vitro and in vivo larva model study, we demonstrate that phages vB_SauM-A, vB_SauM-C, and vB_SauM-D are effective antibiofilm agents. The exposure of biofilm to phages vB_SauM-A and vB_SauM-D led to 2–3 log reductions in the colony-forming unit number in most of the multidrug-resistant *S. aureus* strains. It was found that phage application reduced the formed biofilms independently of the used titer. Moreover, the study demonstrated that bacteriophages are more efficient in biofilm biomass removal and reduction in staphylococci count when compared to the antibiotics used. The scanning electron microscopy analysis results are in line with colony forming unit (CFU) counting but not entirely consistent with crystal violet (CV) staining. Additionally, phages vB_SauM-A, vB_SauM-C, and vB_SauM-D can significantly increase the survival rate and extend the survival time of *Galleria mellonella* larvae.

## 1. Introduction

Many scientists in the last decades have focused their research on designing novel biofilm treatment strategies, as well-known antimicrobial treatment was shown to be inefficient in eradicating biofilms [1]. Tolerance of attached embedded-in-extracellular-polysaccharide-matrix bacterial cells to different disinfectants and antimicrobial agents has been widely researched. There are some indications that they can resist up to 100–1000 times higher concentrations of antibiotics than planktonic cells [2].

A noteworthy example of a bacterium commonly causing biofilm-related infections is the opportunistic pathogen *Staphylococcus aureus*. Staphylococcal biofilms are associated with a broad spectrum of human diseases, such as osteomyelitis, periodontitis, endocarditis, chronic wound infection, and infection of indwelling medical devices [3,4]. Apart from increased tolerance to antibiotics of bacteria in the biofilm, more and more *S. aureus* strains exhibit resistance to the antibiotics previously employed. In addition to being resistant to β-lactam antibiotics (methicillin-resistant *S. aureus*), most strains also become resistant to other antibiotic classes (e.g., fluoroquinolones, macrolides, aminoglycosides, and clindamycin), leading to the emergence of multidrug-resistant *S. aureus* (MDRSA) [4,5].

One of the most promising tools to combat antibiotic-resistant bacteria inside a biofilm are bacteriophages, viruses that specifically lyse bacterial cells [6,7]. Almost completely abandoned, phage therapy is now undergoing a revival, and numerous bacteriophages have been isolated recently [8,9,10]. However, phages destined for antimicrobial therapy applications should be carefully characterized in terms of a life cycle, a spectrum of action, stability, gene content, and interactions between phages and microbial biofilms [11].

Therefore, we aimed to analyze the activity of novel bacteriophages vB_SauM-A, vB_SauM-C, and vB_SauM-D to reduce MDRSA biofilm in vitro and in vivo in a *Galleria mellonella* larva model. Additionally, our study aims to compare phage and antibiotic effectiveness in biofilm eradication.

## 2. Results

### 2.1. MIC Determination

The MIC values of different classes of antibiotics cotrimoxazole (SXT), gentamicin (GM), tetracycline (TE), fusidic acid (FA), and vancomycin (VAN) were determined for 11 MDRSA strains (Table 1). All selected MDRSA strains were susceptible to the selected antibiotics except strains 44 and 70, which were resistant to tetracycline (results published formerly [12]).

### 2.2. Biofilm Eradication by Phages

**Figure 1:**
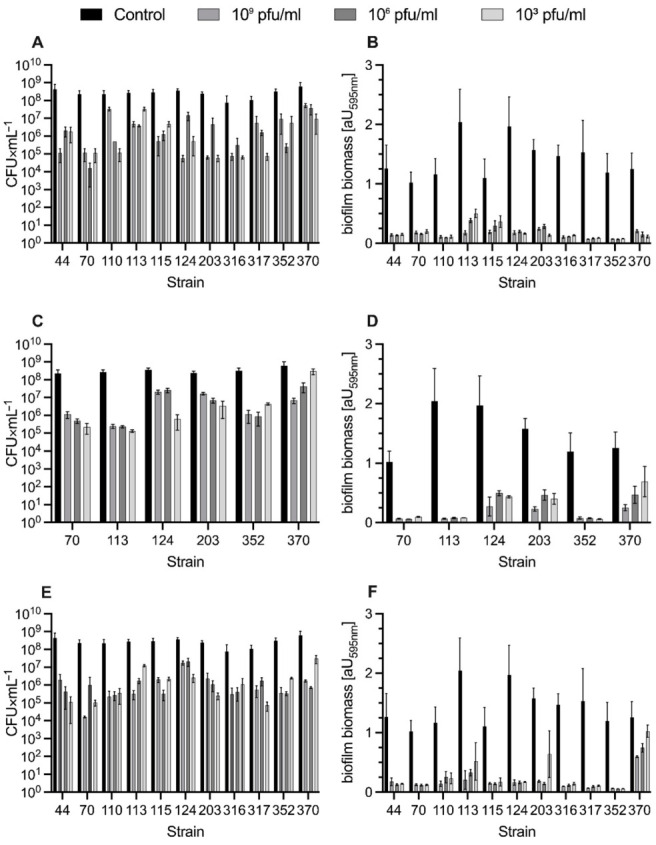
Antibiofilm activity of phages vB_SauM-A (**A**,**B**), vB_SauM-C (**C**,**D**), and vB_SauM-D (**E**,**F**). Effects of phages on the number of viable cells (**A**,**C**,**E**) and biomass reduction (**B**,**D**,**F**).

To evaluate bacteriophage antibiofilm activity, we first selected appropriate MDRSA strains. A strain was chosen for the study only when the following criteria were met: (1) it was characterized as a strong biofilm producer and (2) it was susceptible to phages vB_SauM-A, vB_SauM-C, or vB_SauM-D, which was determined in previous studies [4,12]. Phages vB_SauM-A and vB_SauM-D effectively lysed 11 biofilms producing MDRSA strains, whereas vB_SauM-C lysed only 6 out of 11 [12]. Biofilms treated with 10^3^, 10^6^, and 10^9^ pfu/mL of tested phages after 24 h were assessed for colony-forming unit (CFU) number and total biofilm biomass (CV staining). It was found that phage application reduced the formed biofilms independently of the used titer (Figure 1). When 24-h-old MDRSA biofilm was exposed to vB_SauM-A and vB_SauM-D, the number of adhered bacteria decreased by 2–3 logs in most of the strains tested. The most significant reduction (4.14 log) was caused by phage vB_SauM-D on strain 70 (Figure 1E). The biofilm exposure to vB_SauM-C led to 3 and 2 log reductions in 3 of 6 strains. Statistical analysis reveals that biofilm reduction by bacteriophages was statistically significant compared to the non-treated control.

### 2.3. Biofilm Eradication by Antibiotics

**Figure 2:**
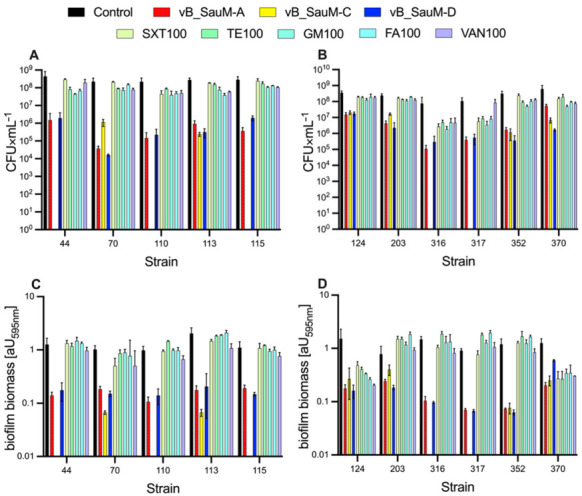
Eradication of biofilms formed by 11 clinical MDRSA strains by phages vB_SauM-A, vB_SauM-C, vB_SauM-D, and five antibiotics: SXT, TE, GM, FA, and VAN. Effects of phages and antimicrobial drugs on CFU number (**A**,**B**), effects of phages and antimicrobial drugs on biomass reduction (CV staining) (**C**,**D**). All values are means of 3 determinations ± SD.

Here, the antibiofilm activity of phages vB_SauM-A, vB_SauM-C, vB_SauM-D, and five antibiotics at concentration 100 × MIC was demonstrated (Figure 2). CFU analysis showed a considerable reduction in staphylococci count in all bacteriophages used compared to control and to the antibiotic treatment group (except the use of vB_SauM-A at strain 370) (Figure 2A,B). Figure 2C,D clearly show that bacteriophages were more efficient in biofilm biomass removal when compared to the antibiotic used. Concerning CV analysis of biofilm biomass, only strains 124 and 370 showed a statistically significant biofilm biomass reduction after antibiotics application. This reduction was similar to reduction caused by bacteriophages (no significant comparison between these groups). Regarding GM and TE use on strains 203 and 317, a statistically significant biofilm biomass increase was observed (Figure 2D).

### 2.4. SEM Analysis of Biofilm

**Figure 3:**
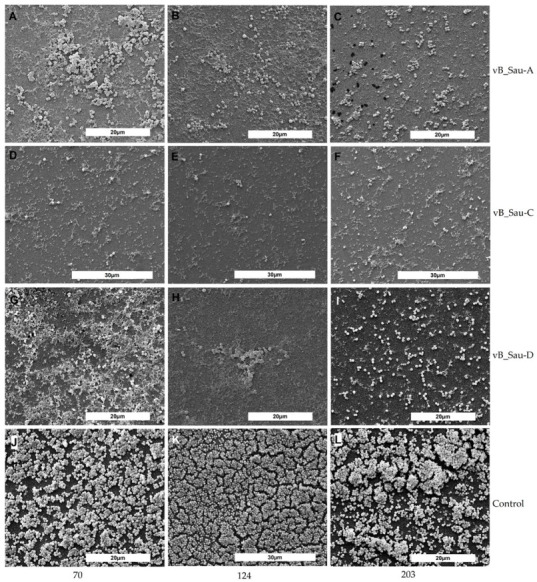
Scanning electron images of biofilms formed by representative MDRSA strains 70 (**A**,**D**,**G**,**J**), 124 (**B**,**E**,**H**,**K**), and 203 (**C**,**F**,**I**,**L**) grown on 6-well plates. Biofilm exposed to bacteriophage vB_Sau-A (**A**–**C**), bacteriophage vB_Sau-C (**D**–**F**), bacteriophage vB_Sau-D (**G**–**I**), nontreated controls (**J**–**L**).

To further confirm our findings, the MDRSA biofilms treated with the phages vB_SauM-A, vB_SauM-C, and vB_SauM-D were visualized by scanning electron microscopy (SEM). Scanning electron micrographs of biofilms formed by representative MDRSA strains are presented below (Figure 3). Biofilm formation was significantly suppressed in all the phage-treated samples. Interestingly, there was more biofilm matrix in the biofilm structure of some strains in the samples treated with phages compared to control (e.g., Figure 3G). The results are in line with CFU counting but not entirely consistent with CV staining.

### 2.5. Galleria mellonella Survival Assay

**Figure 4:**
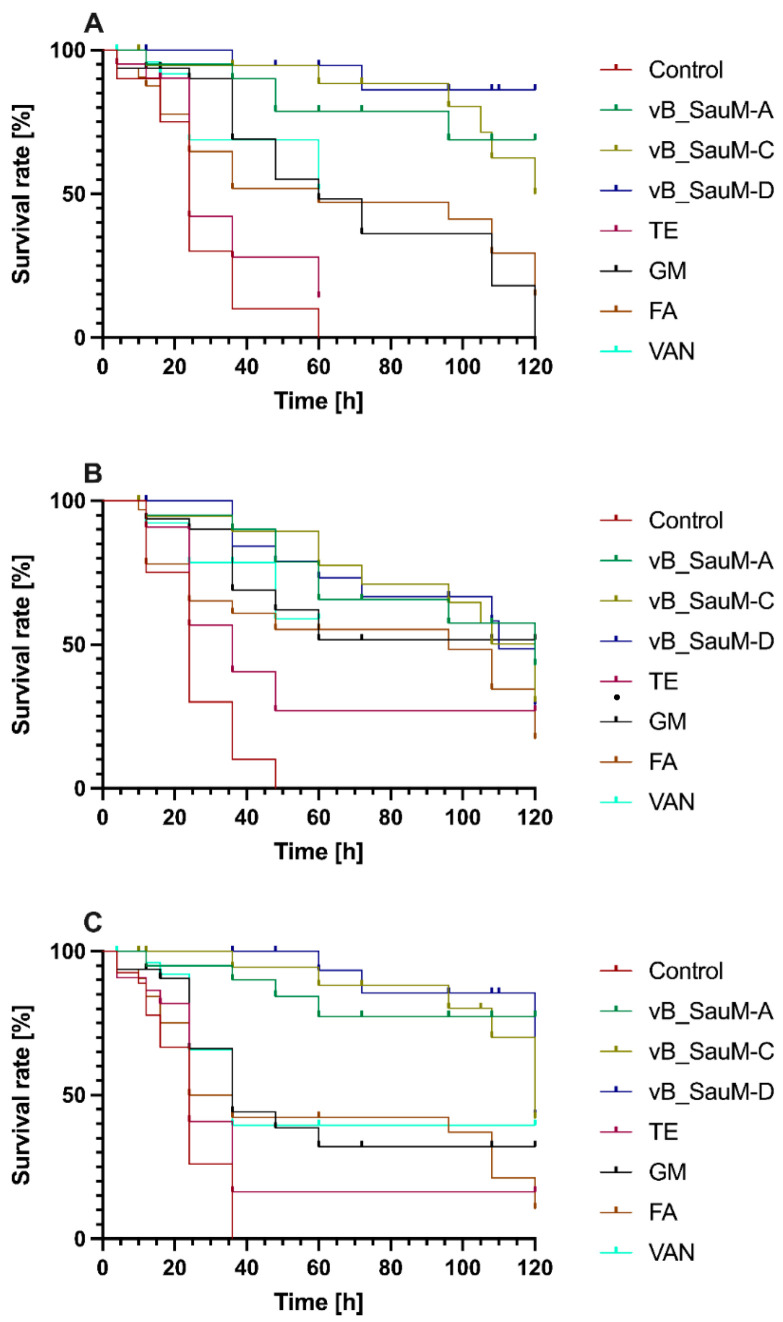
In vivo survival of *Galleria mellonella* larvae after infection with 203 (**A**), 110 (**B**), 352 (**C**) MDRSA strains treated with phages and antibiotics.

To assess the in vivo activity, a wax moth larvae (*Galleria mellonella*) model was used. The in vivo test was performed on three representatives of MDRSA. This model showed the varied effectiveness of all antibiotics used for therapy in the tested strains. Kaplan–Meier analysis with a Mentel–Cox post hoc test revealed no statistically significant differences between antibiotic-treated and non-treated control larvae when strain 203 was used for infection (Figure 4A). In contrast, the use of phage therapy resulted in a statistically significant increase in survival rate and prolonged survival time. The highest increase in survival rate was observed in infected larvae treated with phage vB_SauM-D (control group showed 100% lethality within 60 h, whereas larvae treated with phage vB_SauM-D exhibited 86% of survival after 120 h). In strain 110, antibiotics (excluding tetracycline) and phages showed similar effectiveness, characterized by an increased and prolonged time of larvae survival (Figure 4B). In the case of strain 352, bacteriophages significantly increased larvae survival (Figure 4C). Similarly, in strain 203, the highest increase in survival percentage was caused by phage vB_SauM-D.

## 3. Discussion

Bacterial biofilm is responsible for approximately 80% of infections and poses a high probability of prolonged infection. Additionally, bacteria in the form of biofilm can be up to 1000 times more resistant to antibiotics than the planktonic form. Searching for a novel approach that reduces or eliminates bacterial biofilm is in the medical community’s interest [13,14].

Our study shows that bacteriophages pose a higher ability to reduce and disperse *Staphylococcus aureus* biofilm than antibiotics. All drugs used in this study are commonly employed to treat infections with *S. aureus*, and they were proven to be active against planktonic cells of tested *S. aureus* isolates [15]. As mentioned above, the most chronic and recurrent bacterial infections in humans arise from the formation of a bacterial biofilm. Bacteria in biofilms are 10 to 1000 times more resistant to antibiotics than cells in the planktonic form [13]. Additionally, during therapy, the antibiotic concentration in tissues is lower than the MIC of the drug used [16]. As this study shows, even when a bacterial strain appears to be sensitive to an antibiotic, antibiotic therapy in the presence of biofilm may be ineffective and even increase the biofilm biomass.

There are two fundamental limitations to phage infection of cells inside a biofilm: First, the minimized cell availability for bacteriophages due to the structure of the biofilm and the presence of extracellular material. Therefore, many bacteriophage genomes contain genes for enzymes capable of breaking down elements of the biofilm matrix. Most often, they are small, efficiently diffusing enzymes that target the cell wall of the host bacterium during both initiation of infection and release from the host cell. These enzymes typically also can degrade the exopolysaccharide (EPS) of a biofilm [17,18,19]. The second limitation in the dispersion of biofilms due to bacteriophages is the metabolic state of a part of the population, where surviving cells and bacteria in the stationary phase are characterized by slow metabolism. Most bacteriophages reproduce best using bacteria that grow exponentially. Therefore, bacteria at different metabolic stages can be effectively infected when they enter the growth phase [20,21,22]. The bacteriophages used in this work seem to be much more effective in reducing biofilm than the antibiotics used. Unfortunately, the use of bacteriophages does not completely remove the biofilm under the conditions tested. We also observed that the reduction level does not change significantly with phage concentration, as observed before. Alves et al. (2014) [23] demonstrated that although phage mixtures with higher multiplicity of infection (MOI) gave more rapid reduction in biofilm density, both MOIs, 1 and 10, resulted in the same endpoint after 48 h.

In vitro conditions do not imitate the animal infection models. Referring to this, therapeutic evaluation of the bacteriophage candidate for phage therapy should include ex vivo and in vivo models. Our study demonstrated that *Galleria mellonella* larvae survival was significantly greater in larvae treated with bacteriophages compared to those with antibiotics (in the case of two strains tested). The highest increase in survival rate was observed in infected larvae treated with phage vB_SauM-D for MDRSA strains 203 and 352 and phage vB_SauM-A for strain 110. Earlier, larva models were used to describe phage therapy using different models, both Gram-negative and Gram-positive bacteria [7,24,25,26]. This study shows that the use of bacteriophages poses better therapeutic options than antibiotics used. Tsai et al. (2016) [27] also showed that even though tested bacteria were susceptible to the antibiotics used in in vitro conditions, this does not increase the therapy outcomes of the in vivo model. After injection of phages, antibiotics, and combinations of these factors, the melanization markers of larvae humoral responses were not observed. Accumulated evidence supports the potential of isolated phages for application in therapy [27].

In summary, our study demonstrated that bacteriophages could reduce the biofilm biomass of MDRSA strains more efficiently than antibiotics. There was a 2–3 log reduction in CFU in most cases and a considerable reduction in biofilm biomass. Nowadays, the promising alternative treatment is using bacteriophages and antibiotics together. The use of phage–antibiotics synergy shows promising experimental results [28,29,30,31,32,33]. There is no information about the combined use of Kayviruses and antibiotics, and such information should be completed quickly; thus, our next step is to analyze the synergies between Kayviruses and antibiotics.

## 4. Materials and Methods

### 4.1. Bacterial Strains

A total of 11 multidrug-resistant *Staphylococcus aureus* clinical isolates were chosen from the collection of *S. aureus* strains from the Department of Medical Microbiology, the Medical University of Gdańsk. Strains were differentiated using SCC*mec* and *spa* typing and selected due to their multidrug resistance and ability to form a biofilm, which was studied previously [4,12]. All the strains tested were confirmed to be resistant to β-lactams, erythromycin, norfloxacin, and ciprofloxacin. Moreover, 9 of 11 strains were resistant to clindamycin, 2 of 11 to tetracycline, and 1 of 11 to chloramphenicol.

### 4.2. Phages

Bacteriophages vB_SauM-A, vB_SauM-C, and vB_SauM-D were isolated from different wastewater treatment plants and were previously characterized concerning their morphology, genetics, and biological properties, including host range, adsorption rate, latent time, phage burst size, and lysis profiles [12]. Phage propagation, purification, and enumeration were performed as described previously [12].

### 4.3. MIC Determination

Bactericidal antibiotics of five classes were selected for this study: gentamicin, cotrimoxazole, fusidic acid, tetracycline, and vancomycin, as all MDRSA strains were susceptible to them except strains 44 and 70, which were resistant to tetracycline (results published formerly [12]).

The minimal inhibitory concentration values (MICs) of *S. aureus* isolates to different antibiotics were estimated using E-tests (gentamicin, fusidic acid, tetracycline, and vancomycin) (Argenta, Poznań, Poland) or the standard two-fold dilution protocol (cotrimoxazole) according to the Clinical Laboratory Standards Institute (CLSI) [34].

All antibiotics used for biofilm eradication were obtained from Sigma–Aldrich. Stock solutions of the antibiotics were prepared in sterile distilled water to a final concentration of 10 mg/mL except cotrimoxazole, which was initially prepared using a 1:5 mixture of trimethoprim: sulfamethoxazole in dimethyl sulfoxide (Sigma–Aldrich, Saint Louis, MO, USA). Working stocks of these antibiotics were prepared in Mueller Hinton broth (Biomaxima, Lublin, Poland) [3].

### 4.4. Biofilm Eradication

Biofilms were formed on 24-well polystyrene microtiter plates using the protocol described before [12] with minor modifications. Each well was inoculated with 500 µL of this bacterial suspension, and the microtiter plates were incubated for 24 h at 37 °C. After incubation, established biofilms were washed twice with PBS, and 500 µL of phage or antibiotic mixture in LB was added to a set of wells. Three different titers were set up for the phage, 10^3^, 10^6^, and 10^9^ pfu/mL. Antibiotics were added at the MIC for planktonic cells and at 100 × MIC. All the experiments were performed three times. After static incubation at 37 °C, microplates were washed twice with PBS, and biofilm eradication was assessed with colony-forming unit (CFU) quantification and crystal violet staining.

The crystal violet (CV) assay was applied to assess the amount of total biofilm biomass. Briefly, the biofilm layer was fixed with methanol and stained with 1% crystal violet for 15 min. Excess stain was rinsed off by running tap water until the water was colorless, and the plate was left to air dry. To solubilize the dye bound to the biofilm, 200 µL of ethanol-acetic acid-water (30:30:40) was added to the wells, and the optical density at 570 nm was measured in the microplate spectrophotometer.

The number of bacteria adhered to the surface of microplate wells was enumerated as described by Cruz et al. [35] with some alterations. Therefore, 500 µL of PBS was added to each well, and biofilm cells were suspended by vigorous pipetting. The 10-fold serial dilutions were immediately performed in 0.9% saline solution, and 40 μL of the dilutions were directly plated on LB plates.

### 4.5. Microscopic Examination of Biofilms

Biofilm for scanning electron microscopy (SEM) analysis was performed on 6-well polystyrene plates (PPHU Genos s.c., Strońsko, Poland) following the method described at point 4.4. After 24 h of incubation with phages or antibiotics, the wells were washed with sterile PBS to remove all planktonic cells. After washing, adhering bacterial cells were fixed with 3% buffered glutaraldehyde (Poch S.A., Gliwice, Poland) overnight at 4 °C as described before [36]. The samples were dehydrated through a graded ethanol (Poch S.A., Gliwice, Poland) series (50%, 70%, 80%, and 90%) for 10 min each and two times with 96% for 30 min at room temperature. The bottom of each well was cut to a length suitable for electron microscopy. After coating with a 10-nm-thick conductive layer of gold deposited by a high-vacuum sputter coater (LEICA EM SCD 500, Leica Microsystems, Wetzlar, Germany), the samples were examined with a scanning electron microscope (QUANTA FEG 250, FEI, Hillsboro, OR, USA) using a secondary electron detector (ETD).

### 4.6. Phages and Antibiotics Effectiveness in an In Vivo Model—Galleria mellonella

Larvae of *Galleria mellonella* characterized with cream color and weight of 300 mg were chosen for the experiments. The procedure of the in vivo test was performed based on Szymczak et al. (2020) [24]. Firstly, *G. mellonella* larvae were infected with proper strain of *S. aureus* (approx. 10^5^ CFU/mL) into the larval hemolymph behind the last proleg. Then, after 20 min of incubation at room temperature, larvae were subjected to the curation with one of the antibiotics (gentamicin, fusidic acid, tetracycline, or vancomycin) or one of the phages (vB_SauM-A, vB_SauM-C, or vB_SauM-D). The concentration of antibiotics was equal to MIC value and was calculated based on the average weight of larvae, and suspension of bacteriophages was at MOI = 1. The injection was made on the opposite side to the bacterial injection. Larvae were incubated at 37 °C and examined every 12 h. Larvae that exhibited dark color and did not respond to physical contact were considered dead. Untreated larvae were considered a control group.

### 4.7. Statistical Analysis

All experiments were conducted with a minimum of five biological replicates. Technical replicates were averaged to produce replicate means that were used for analysis. Mean values were compared using one-way ANOVA followed by Tukey post hoc test for pairwise comparisons. Differences were considered statistically significant if *p* < 0.05. To analyze *G. mellonella* survival, the Kaplan–Meier survival test, followed by the Mantel–Cox test, was performed. All statistical analyses were carried out using GraphPad Prism 9 (Graph Pad Software, San Diego, CA, USA).

## 5. Conclusions

In conclusion, our results suggest that phages vB_SauM-A, vB_SauM-C, and vB_SauM-D are more efficient in biofilm removal compared to the antibiotics used in the experiment, especially when biofilm biomass is analyzed. Moreover, using an in vivo *G. mellonella* larva model, we demonstrated that bacteriophages have a positive effect on the survival of organisms infected with MDRSA strains. Therefore, we conclude that the bacteriophages we tested can be a promising tool for potential use in the treatment of MDRSA infections, but further investigations on mammalian models are necessary.

## Figures and Tables

**Table 1:** The MIC values of cotrimoxazole (SXT), gentamicin (GM), tetracycline (TE), fusidic acid (FA), and vancomycin (VAN) for 11 MDRSA strains.

MDRSA Strain	MIC Value [µg × mL^−1^]				
	SXT	GM	TE	FA	VAN
44	2.048	0.38	R	0.188	1.5
70	1.024	0.38	R	0.188	1.5
110	2.048	0.25	0.094	0.125	1.5
113	2.048	0.19	0.094	0.188	1
115	4.096	0.25	0.094	0.094	1.5
124	2.048	0.19	0.094	0.125	1
203	2.048	0.19	0.094	0.125	1.5
316	2.048	0.76	0.375	0.375	1.5
317	2.048	0.76	0.375	0.188	1.5
352	2.048	0.38	0.375	0.188	1.5
370	2.048	0.38	0.094	0.094	1.5

## Data Availability

The data presented in this study are available on request from the corresponding author.
